# Microplastics in Spanish Table Salt

**DOI:** 10.1038/s41598-017-09128-x

**Published:** 2017-08-17

**Authors:** Maria E. Iñiguez, Juan A. Conesa, Andres Fullana

**Affiliations:** Department of Chemical Engineering, University of Alicante, P.O. Box 99, 03080 Alicante, Spain

## Abstract

Marine debris is widely recognized as a global environmental problem. One of its main components, microplastics, has been found in several sea salt samples from different countries, indicating that sea products are irremediably contaminated by microplastics. Previous studies show very confusing results, reporting amounts of microparticles (MPs) in salt ranging from zero to 680 MPs/kg, with no mention of the possible causes of such differences. Several errors in the experimental procedures used were found and are reported in the present work. Likewise, 21 different samples of commercial table salt from Spain have been analyzed for MPs content and nature. The samples comprise sea salts and well salts, before and after packing. The microplastic content found was of 50–280 MPs/kg salt, being polyethylene-terephthalate (PET) the most frequently found polymer, followed by polypropylene (PP) and polyethylene (PE), with no significant differences among all the samples. The results indicate that even though the micro-particles might originate from multiple sources, there is a background presence of microplastics in the environment.

## Introduction

Marine debris (MDs) are widely recognized as a global environmental problem^1–3^. They produce a wide range of negative effects, not only environmental, but also economical, safety, health and cultural impact.

The materials which are most commonly found in marine debris comprise glass, metal, paper and plastic^4^; being plastic debris the most abundant in the marine environment^4–8^. They represent between 60 and 80% of the total marine debris^9^.

Plastics are relatively recent, they have existed only for around one century^10^, but since the development of the plastics industry, plastic items have spread around the world. Recent studies estimate that between 4.8 and 12.7 million tons of plastic waste ends up in the world’s oceans every year^2^, making them the largest plastic dumps.

Plastics are harmful to the marine environment, mainly because of their resistance to degradation. Once in the environment, and especially in the marine environment, the decomposition of plastic items occurs in an exceedingly long time, usually estimated between hundreds and thousands of years^11^. Furthermore, during this time, plastics are fragmented into small pieces, becoming plastic micro-particles (with a diameter of less than 5 mm)^12^.

Many products of human consumption, such as salt, fish… come from the sea. Marine pollution can affect these products, and microplastics can reach our organism through them. Recent studies^13^ show the presence of plastic microfiber in deep-sea organisms, showing that the deep sea is already worryingly exposed to human waste.

There are several types of table salt according to their origin: sea salt, lake salt, rock salt and river or well salt. Sea salt and lake salt are obtained by evaporation, rock salt comes from the mining of a mineral rock called halite, and river or well salt is obtained from wells in non-coastal zones. Sea salt and rock salt are the most sold and consumed in Spain.

**Figure 1:**
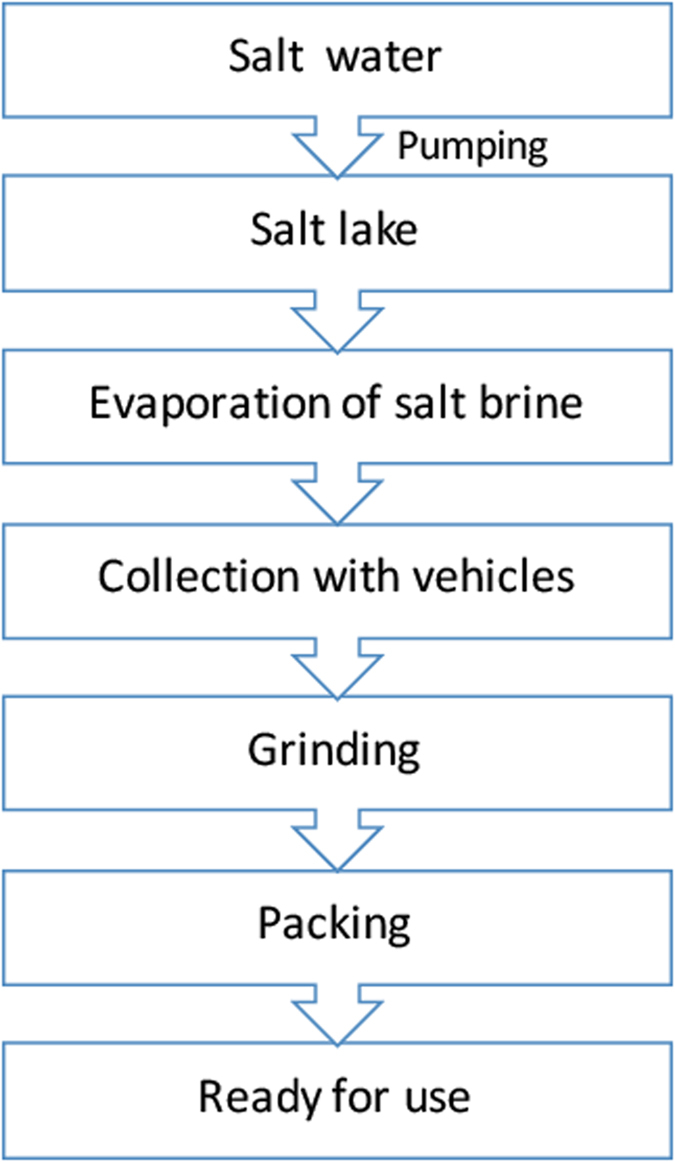
Scheme of the salt manufacturing process.

In the production of sea or lake salt, saltwater is pumped into evaporation ponds, where it is concentrated by the action of sun and wind. After that, the salt condenses and crystallizes on the surface of the crystallizers, where the salt is cut and collected by means of a controlled collection process. Subsequently, salt undergoes different physical processes before its packing in different containers for their multiple uses and different applications. Figure 1 shows a scheme of the salt production process. For manufacturing river or well salt, the saline water is pumped from below ground into salt lakes, where it concentrates similarly to sea salt.

Recent studies showed that microplastics (fragments, fibers and pellets) are present in seas all over the world^14–16^, although only microfibers (not microbeads) where found in deep-sea organisms^13^. This lead to the hypothesis that sea salt might contain microplastics in it, because it is directly produced from seawater^17^, pointing out the importance of monitoring the presence of such contaminants in sea salts.

In the work of Yang *et al*. the amounts of microparticles (MPs, comprising fragments, fibers and pellets) found in different Chinese salts are in the range 7–680 MPs/kg. On the contrary, an study recently published in Scientific Reports by Karami *et al*.^18^ concluded that the amount of MPs in salt of different origins is nil, in the range 0–10 MPs/kg, without a comparison with previous studies. Surprisingly, the authors of the paper^18^ are trying to retain microfibers of a size of between 10 and 200 μm, approximately, in a filter whose pore size is 150 μm. This result is that the amount of fibers detected is extremely low, as most are expected to pass through the filter. Previous studies by Yang *et al*.^17^ mention amounts of fibers around 700 microparticles MP/kg (using a 5 μm pore sized filter), while the amounts detected in this case are 0–10 MP/kg. The authors specifically cite the work of Yang *et al*., but do not even compare their results nor mention of the differences found. By contrast, Karami *et al*. perform a validation of the experimental method with model samples, but in this case they used a filter whose pore size is much smaller, 8 μm.

With the aim of designing a veritable method to isolate MPs and check it with samples from the Spanish salt market, in the present work several types of salt produced in different points of Spain have been studied. Marine and well salt have been collected to compare both types. Also the effect of the packaging process is discussed. The abundance and nature of the microplastics found have been analyzed by means of stereo microscopy and Fourier Transform Infrared Spectroscopy (FT-IR).

## Results and Discussion

### Number of microfibers

**Figure 2:**
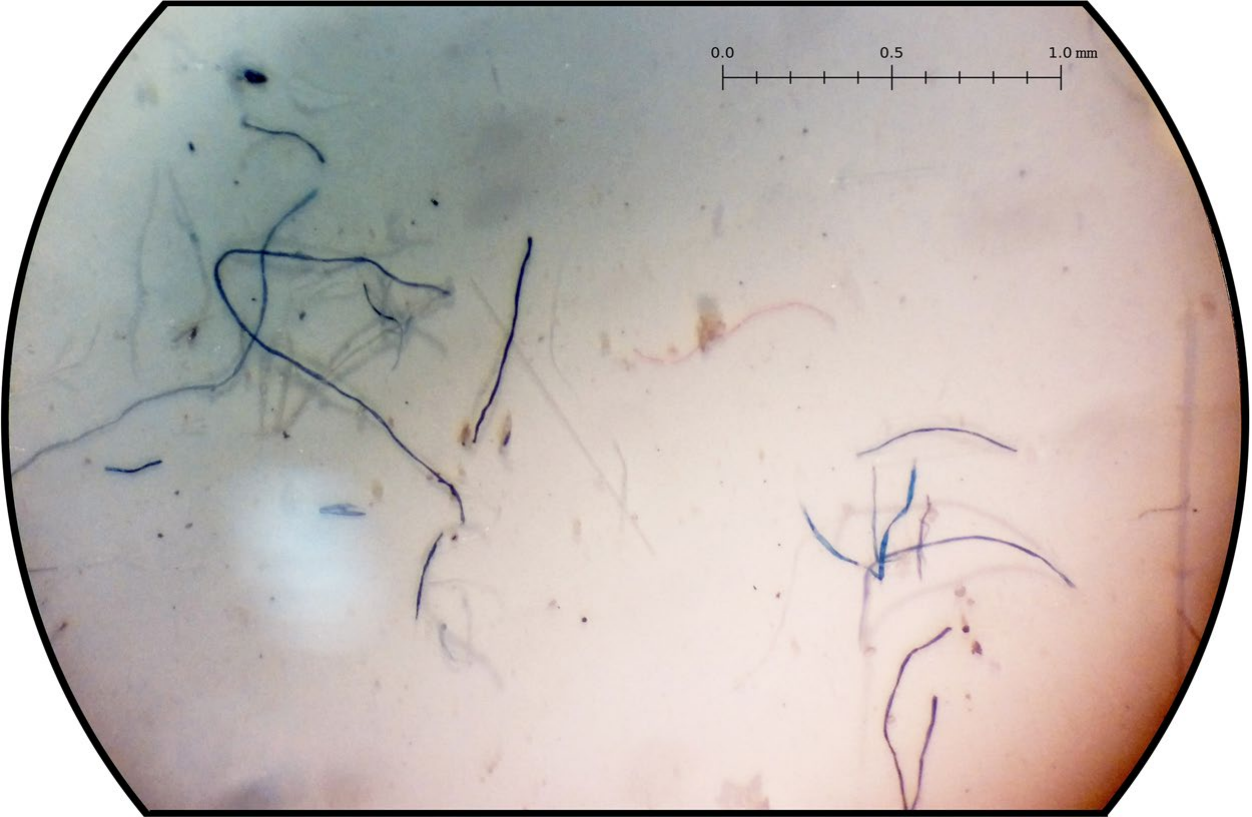
Optical microscope image of a filter after the filtration of a salt sample (The target used was 3.0×).

As a representative example, a photograph of one of the filters is shown in Fig. 2. The size of plastic fibers ranged from 30 μm to 3.5 mm in all table salt samples. No fibers smaller than 30 µm were found. The most common colors found were black, red, blue, white and transparent.

The number of particles/kg in the different types of salt is summarized in Table 1 (analysis was triplicated and confidence intervals are given, data were analyzed using the fixed-effects model of analysis of variance). As can be seen, different brands presented no significant differences in the abundance of microplastics in marine salts. There is also no significant difference between the amount of fibers found in well salt samples (ranging from 115 to 185 particles/ kg) and sea salt samples (from 50 to 280 particles/ kg).

**Table 1:** Number of microplastics found in the different types of table salt analyzed.

	Source	Type of salt	Origin	N° particles /kg
Sample 1	Sea salt	coarse	Atlantic Ocean (Huelva)	120 ± 7
Sample 2	Sea salt	fine	Atlantic Ocean (Huelva)	140 ± 3
Sample 3	Sea salt	fine	Atlantic Ocean (Huelva)	150 ± 10
Sample 4	Sea salt	fine	Atlantic Ocean (Cádiz)	100 ± 3
Sample 5	Sea salt	coarse	Atlantic Ocean (Lanzarote)	95 ± 7
Sample 6	Sea salt	coarse	Atlantic Ocean (La Palma)	140 ± 8
Sample 7	Sea salt	fine	Atlantic Ocean (Galicia)	50 ± 7
Sample 8	Sea salt	fine	Mediterranean Sea (Barcelona)	190 ± 7
Sample 9	Sea salt	fine	Mediterranean Sea (Barcelona)	80 ± 3
Sample 10	Sea salt	fine	Mediterranean Sea (Gerona)	120 ± 3
Sample 11	Sea salt	fine	Mediterranean Sea (Valencia)	115 ± 10
Sample 12	Sea salt	coarse	Mediterranean Sea (Valencia)	65 ± 7
Sample 13	Sea salt	fine	Mediterranean Sea (Alicante)	175 ± 10
Sample 14	Sea salt	fine	Mediterranean Sea (Murcia)	280 ± 3
Sample 15	Sea salt	fine	Mediterranean Sea (Murcia)	105 ± 7
Sample 16	Sea salt	coarse	Mediterranean Sea (Menorca)	60 ± 10
Sample 17	Well salt	coarse: without grinding		115 ± 10
Sample 18	Well salt	fine: unpacked	Underground river in Alicante, 60 km away from the sea	185 ± 3
Sample 19	Well salt	fine: packed		120 ± 7
Sample 20	Well salt	coarse	Cuenca, 170 km away from the sea	135 ± 7
Sample 21	Well salt	fine	Añana, 60 km away from the sea	140 ± 3

.

From the data presented in Table 1, when comparing the samples 1 (before grinding) and 2 (after grinding), both from the same manufacturer, it can be concluded that the grinding process does not influence the amount of microplastics found in these table salts. For the well salt samples, no important differences were found in relation to the grinding process either, i.e. between the sample 17 (before grinding) and 18 (after grinding).

In the study, packed and un-packed salt of the same well have also been analyzed (samples 18 and 19), in order to assess the influence of the packing process. Table 1 shows that the origin of the microplastic is not related to the packaging process, since a similar microplastic content was found before and after this process.

### Microfiber characterization

The identification of the fibers found was done by Fourier Transform Infrared Spectroscopy (FT-IR), which is one of the most popular methods used to confirm the composition of microplastics^19^.

**Figure 3:**
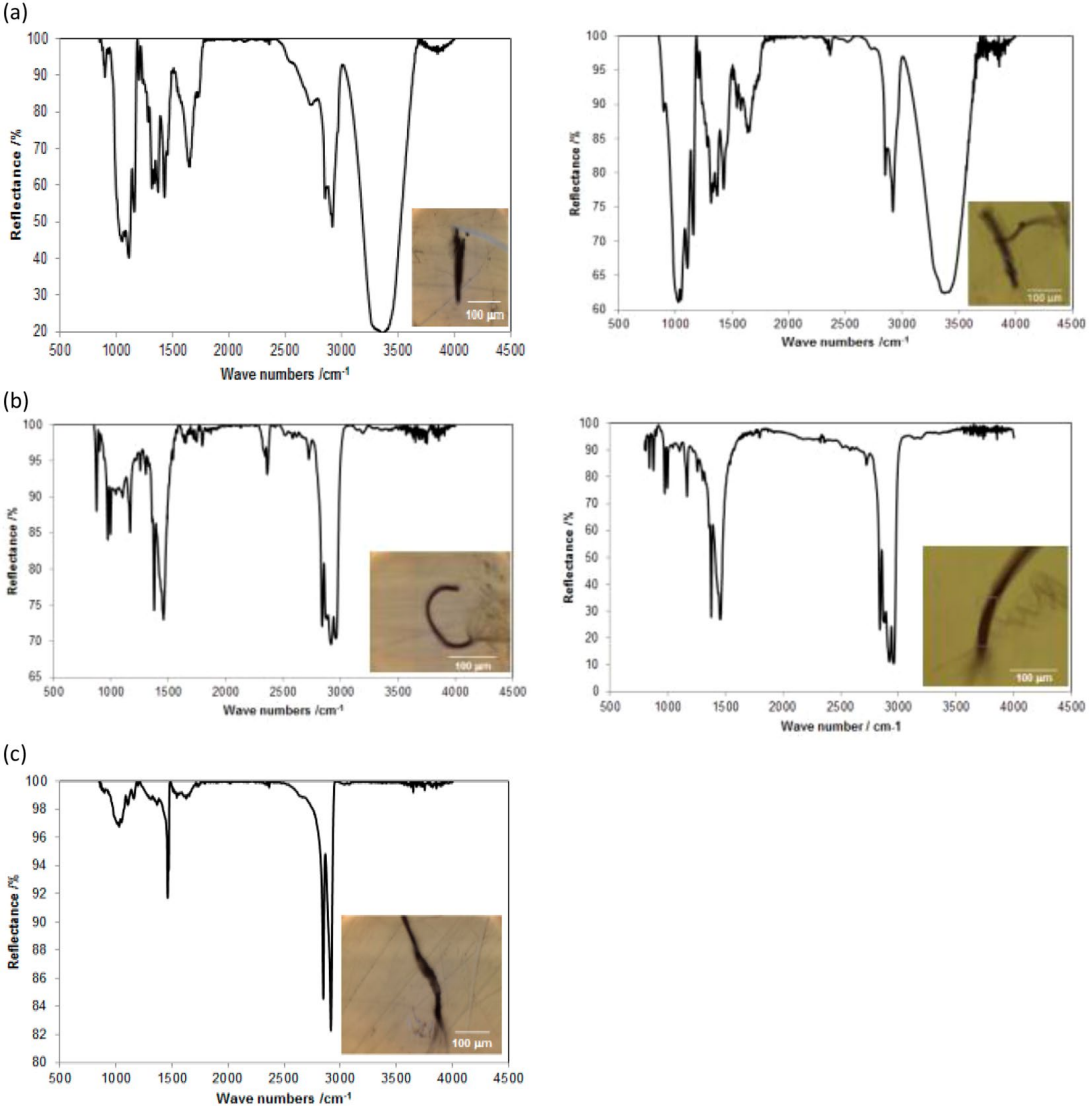
FTIR spectra of fibers found in the table salt samples: (**a**) Two PET fibers; (**b**) Two PP fibers; (**c**) A PE fiber.

Using this technique, several types of microplastics were identified, being the most common ones polyethylene terephthalate (PET), polyethylene (PE) and polypropylene (PP), with a presence, respect to the total of fibers analyzed, of 83.3%, 3.3% and 6.7%, respectively. The remaining 7% corresponds to other particles that have not been identified. Figure 3a–c shows the spectrum of some of the main types of fibers analyzed. In each case, two spectra are shown for fibers found in different samples, in order to compare them. As can be seen, the spectra are very similar, and the identification is definite.

The abundance of the different plastics is logical, since the most commonly used plastics in the world are polyethylene, polypropylene and polyethylene-terephthalate; therefore, they are the most frequently found in the marine environment too^3,20–22^. However, the relative abundance of PET is very high, accounting for 83.3% of the analyzed fibers. Yang *et al*. suggested that this might be due to the high density of PET (1.30 g/cm^3^) in comparison to PE (0.94 g/cm^3^) and PP (0.90 g/cm^3^), which causes it to remain with the salt during the crystallization process in the salt production.

PET is a material which is widely used in packaging, either flexible (as in plastic films) or rigid (as in bottles). PET is also the most used polyester in the textile sector, hence it is in the form of fibers in the sea and in the global environment.

Although experimental methods are not exactly the same, the amount of fibers found in the present study should be compared with the work of Yang *et al*., in which the table salt came from supermarkets throughout China^23^. Table 2 shows the comparison of some data from both studies. The results found by Yang *et al*. indicate that the number of microplastics found in Chinese salts is much higher than the one in the present study, almost double in the case of sea salts; these authors also pointed out remarkable increase in the amount of fibers found in salt which come from the marine environment. In this way, Yang *et al*. indicate that the abundance of microplastics in sea salt was significantly higher than in lake and rock/well salts. However, this result is not found in the present work, being the microplastics content very similar in salt coming from marine and non-marine environments. This should be related with the pollution level and the plastic presence in the Spanish and Chinese seas. Eriksen *et al*.^24^ report an estimate of the total number of plastic particles in the world’s ocean, indicating a very high plastic pollution in north Pacific compared to Mediterranean sea. Yang *et al*.^23^ indicate that in China the sources of sea salts were from coastal waters in locations where the population density is very high, not being this true in the present study. In the present case, the data indicate that there is not a clear source of these micro-particles, but there is a background presence of the microplastics in the environment. In line with this, microplastic pollution has also been detected in honey and sugar samples^25^ and other^26^, as well as in marine organisms^27,28^.

**Table 2:** Comparison of the results of the present study with Yang *et al*. (2015) study on Chinese salts.

	Number of particles/kg		% PET	% PP	% PE	% CP^*^
	Sea salt	Well/Rock Salt				
Spanish Salt	50–280	115–185	83.3	6.7	3.3	0
Chinese Salt	550–681	7–204	13.8	3.9	7.2	33.6

^*^Cellophane (CP).

As mentioned before, the work published by Karami *et al*.^18^ found amount of MPs in salts in the range 0–10 MPs/kg, but an experimental error made this result unessential.

### Microplastics in food and risk to human health

Marine debris produces a wide variety of negative environmental, economic^29^, safety, health^30^ and cultural impacts^11^. The presence of microplastics in the sea water has been revealed as hazardous. In literature, three possible toxic effects of plastic particle have been indicated: first due to the plastic particles themselves, second to the release of persistent organic pollutant adsorbed to the plastics^31^, and third to the leaching of additives of the plastics^26^. Persistent organic pollutants (POPs) such as polychlorinated biphenyls (PCBs) and organochlorine pesticides are present in aquatic systems worldwide. Coastal environments contain keystone species and are a source of food supply^32^. Plastics might absorb these contaminants from the seawater and transfer them to the sea products (fish, salt,..). So the presence of plastics in the sea salt might pose a threat to food safety. Different studies have addressed the sorption capacity of plastic debris for POPs^33–36^. For this, authors such as Seltenrich and Bouwmeester highlighted in their research^26,37^ the importance of investigating the risk of the transmission of these microplastics from the food chain to humans.

According to the World Health Organization (WHO), the maximum salt intake in an adult should not exceed 5 g per day. In this way, an average consumer of Spanish salt would ingest a maximum of approximately 510 plastic particles each year. However, microplastics can reach the human organism through other type of food from sea, such as fish and mussels. There are several studies on ingestion of plastic by fish in the field^38–41^. Foekema *et al*. concluded in a recently research that more than 80% of the fish that ingested plastic contained only one particle, suggesting that microplastics do not seem to remain over a long period of time inside the gastrointestinal tract of fish^42^. However, the presence of microplastics is higher in mussels^43,44^, reaching concentrations of up to 178 microfibres per mussel^45^. So if you compare our result with the intake of a person’s microplastics through another type of food from the sea, such as fish and mussels, it can see that 510 particles/year is not a big quantity of microplastics.

## Method and Materials

**Figure 4:**
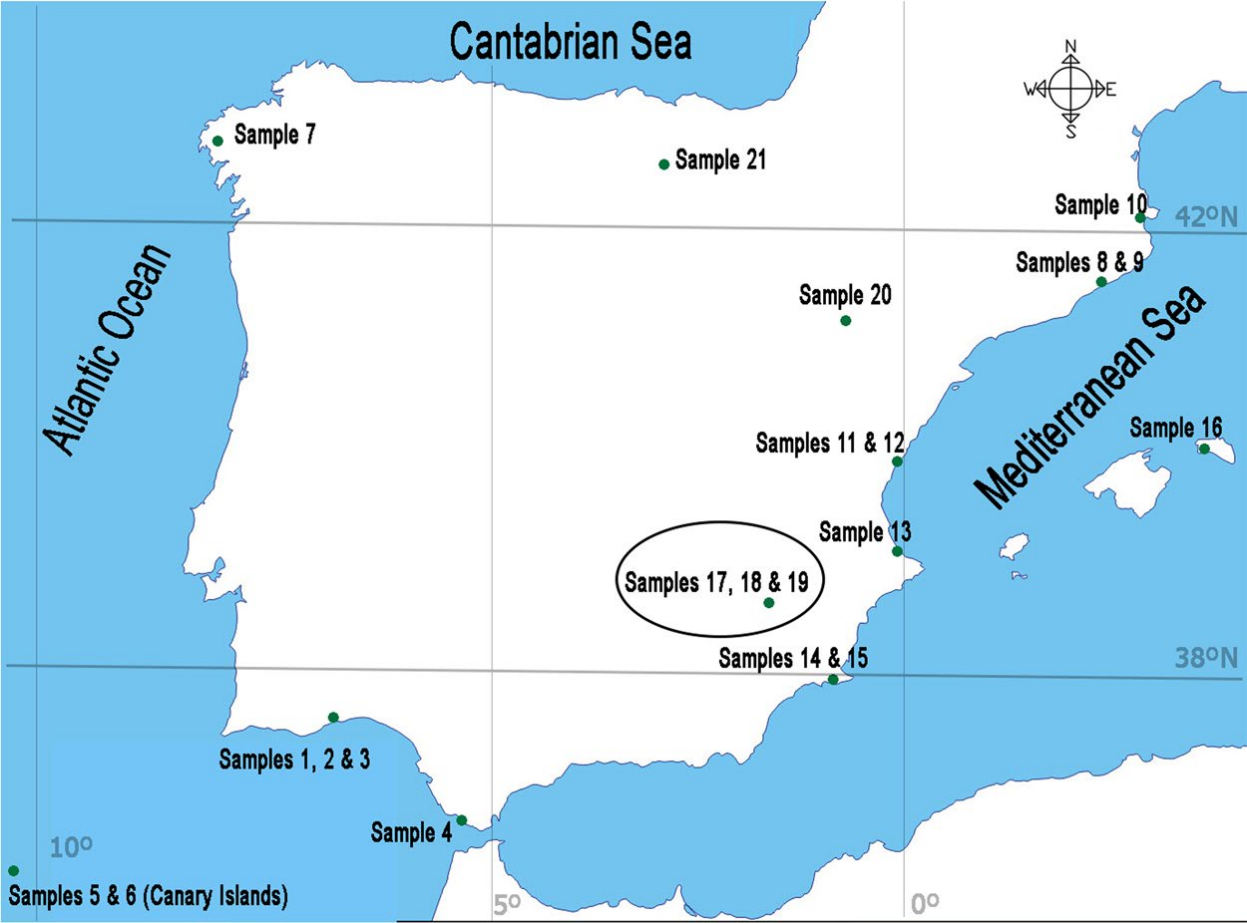
Map of Spain showing the sampling points. This Figure was done using Photoshop CS6 and was created modifying an image freely available at the following web: http://d-maps.com/carte.php?num_car=2190&lang=es. The maps on this page are free for any use, even commercial.

Twenty-one different table salt samples were collected for the present study, all of them from Spanish salt producers, during September 2016–June 2017. An average package with a weight of 1 kg was chosen. All samples were collected from supermarkets, and all of them corresponded to salts manufactured in saltworks along Spain. Figure 4 details the origin of each of the salt samples. Note that some of the samples (samples 17 to 21) are coming from salt producers not located in the coast, i.e., these samples are well salts. As described in Table 1, samples of different particle size were used. Also samples were taken after and before packaging, in order to check for possible differences. Mediterranean Sea and Atlantic Ocean are the coastal areas of study.

As remarked before, two brand of well salt were also analyzed, but collecting separate samples at different stages of the manufacturing process: before grinding, before packing and once packed (see scheme in Fig. 1), corresponding to samples 17, 18 and 19, respectively. This was done in order to elucidate the possible origin of the microplastics. Note that the well salt is also concentrated in salt lakes by evaporation, and collected by vehicles, but its origin is not marine.

Approximately 200 g of each type of salt were dissolved in 1 L of distilled water. The density of this mixture was determined (by using a pycnometer) and give an average value of 1.27 g/cm^3^. Then, the solution was placed in a centrifuge at 1900 rpm for 1 h, in order to separate the possible sand contained by the salt from the saline solution. Although the density of PET is quite similar to that of the saline solution (ca. 1.3 g/cm^3^), the fact that the plastics are in form of microfibers highly decreases their apparent density^46^ so all MPs are expected to remain at the upper part of the solution. That solution was immediately filtered through a piece of 5 μm pore size, 47 mm cellulose nitrate filter paper using a vacuum system^23^.

Once the solution had been filtered, the filter paper was placed into a clean Petri dish with a cover and was dried at room temperature, to later examine the total number of particles under a microscope. This procedure was repeated in triplicate for each sample, using salt from the same package.

The filters were observed under a Leica S6 D Stereozoom CLS150X microscope (range of magnification 0.63× – 4.0×), under polarized light. The amount of microplastics was determined by manually counting the particles contained in the filters.

In order to rule out the possibility of contamination during the sample handling process, a blank measurement was performed. The procedural blank only contained 6 particles of microplastics per filter.

Some fibers of the fibers found on the filters were randomly selected for analysis using FT-IR^21,23^ in a Microscope FTIR JASCO IRT-5200 with 16x Cassegrain lens and MCT detector (7000 − 600 cm^−1^). All samples were measured in transmission. The spectrum range was set to 850–4000 cm^−1^. The spectral resolution was 4 cm^−1^ for all samples and the aperture size varied widely depending on the size of the fibers. All the spectra obtained were compared to the library to identify the polymer type (NICODOM IR Libraries). The spectrum analysis followed the method of Woodall *et al*.^28^.

### Data Availability

The datasets generated during and/or analyzed during the current study are available from the corresponding author on reasonable request.
