# Author Correction: Microplastics in Spanish Table Salt

**DOI:** 10.1038/s41598-018-23060-8

**Published:** 2018-04-12

**Authors:** Maria E. Iñiguez, Juan A. Conesa, Andres Fullana

**Affiliations:** Department of Chemical Engineering, University of Alicante, P.O. Box 99, 03080 Alicante, Spain

Correction to: *Scientific Reports* 10.1038/s41598-017-09128-x, published online 17 August 2017

In this Article, the authors misinterpreted the data previously presented by Karami *et al*. (Ref 18).

In the Introduction section,

“On the contrary, an study recently published in Scientific Reports by Karami *et al*.^18^ concluded that the amount of MPs in salt of different origins is nil, in the range 0-10 MPs/kg, without a comparison with previous studies. Surprisingly, the authors of the paper^18^ are trying to retain microfibers of a size of between 10 and 200 μm, approximately, in a filter whose pore size is 150 μm.”

should read:

“On the contrary, a study recently published in Scientific Reports by Karami *et al*.^18^ concluded that the amount of MPs in salt of different origins is nil, in the range 0-10 MPs/kg, but only MPs of >149 μm are considered.”

In the Results and Discussion section under the subheading ‘Microfiber characterization’,

“As mentioned before, the work published by Karami *et al*.^18^ found amount of MPs in salts in the range 0-10 MPs/kg, but an experimental error made this result unessential.”

should read:

“As mentioned before, the work published by Karami *et al*.^18^ found amount of MPs in salts in the range 0-10 MPs/kg, but experimental differences make the comparison difficult.”

